# An Examination of Peer Victimization and Internalizing Problems through a Racial Equity Lens: Does School Connectedness Matter?

**DOI:** 10.3390/ijerph18031085

**Published:** 2021-01-26

**Authors:** Danielle R. Eugene, Jandel Crutchfield, Erica D. Robinson

**Affiliations:** School of Social Work, University of Texas at Arlington, Arlington, TX 76019, USA; jandel.crutchfield@uta.edu (J.C.); erica.robinson@mavs.uta.edu (E.D.R.)

**Keywords:** ethnic minority youth, peer victimization, mental health, school connectedness, protective factors

## Abstract

Although research has given ample consideration to the association between peer victimization and internalizing problems, little is known about the mediating and moderating influences on this relationship. This study investigated whether peer victimization at age 9 indirectly related to internalizing problems at age 15 via school connectedness and whether the direct and indirect associations between peer victimization and internalizing problems were moderated by race. Data were drawn from the Fragile Families and Child Wellbeing Study, which included 2467 adolescents. The sample was equally divided between male and female and 82% identified as Black and Hispanic. Results indicated that the predictive effect of peer victimization over a 6-year period on teen depression and anxiety was explained by increased school connectedness. Furthermore, there was a moderating effect of race on the direct effect of school connectedness and teen depression and anxiety. For both White and ethnic minority youth, increased school connectedness was associated with less teen depression and anxiety. However, this effect was weaker for ethnic minority students in comparison to White students in both moderated mediation models. The moderated mediation results for teen anxiety showed a greater differential effect among race. The findings have important implications, which are discussed.

## 1. Introduction

During their lifetime, many youth experience internalizing mental disorders with onset peaking during early adolescence [1]. In a recent study, researchers found that one in 20 youth in the United States had a diagnosis of depression or anxiety [2]. Depression is commonly described as persistent sadness, or an irritable mood and anxiety is often referred to as excessive fear or worry [3]. These conditions have been associated with negative psychosocial outcomes including disengagement in school, substance misuse, risky sexual behaviors, and increased suicide risk [4]. Beyond this, these internalizing problems frequently persist into adulthood and are associated with an increased risk of co-occurring disorders, lower wage earnings, and early death [5].

Although all youth are vulnerable to mental health problems, those who encounter traumatic experiences are at greater risk [6]. Particularly, youth who are victims of bullying. Peer victimization is prevalent among school-age youth and has been found to contribute to internalizing mental disorders [7]. Much of this research focuses on one developmental period, middle childhood, leaving a notable gap in the field’s understanding of development and long-term outcomes. According to the life course perspective, development is lifelong and no life stage can be understood in isolation from others. Therefore, adolescent mental health outcomes can be fully understood in light of previous childhood experiences [8]. Moreover, even less is known about the contextual factors that may contribute to the link between peer victimization and internalizing symptomology, which can directly inform early prevention and intervention efforts [9]. School connectedness is a salient factor for school-age youth and may contribute to how strongly peer victimization is associated with internalizing symptoms [10,11,12]. Accordingly, the current study extends previous research by investigating whether peer victimization is associated with symptoms of depression and anxiety among adolescents over a 6-year period, and whether school connectedness mediates this link. In addition, this study explored whether this mediation process was moderated by race.

## 2. Literature Review

### 2.1. Peer Victimization and Internalizing Problems

While the United States is gripped in a fierce public health crisis in the COVID-19 pandemic, it also faces the sustained public health crisis of bullying and peer victimization. Bullying, peer victimization, and their relationship to internalizing problems has been given ample space within the literature. The concept of bullying has long been studied and is often characterized as physical, emotional, or relational (non-sexual) harm done to others [13]. The physical component might involve hitting, kicking, or punching, while the emotional component might involve teasing or disparaging remarks. Relational bullying involves one individual trying to cause harm to another’s reputation by spreading rumors or damaging relationships so that a victim feels isolated. This is also referred to as indirect aggression as opposed to direct aggression involving physical bullying. Peer victimization is most commonly defined as repeated exposure to these negative actions for some time with a power imbalance between the bully and the victim [14,15,16]. Bullying and peer victimization also can occur online and through text messages, which is known as cyberbullying. With the rise of distance learning during this pandemic, more focus has been given to cyberbullying. Upwards of 20% of high school students were bullied on campus and another 15% were bullied online [17]. Within each of these areas of bullying, children and adolescents experience peer victimization whether physical, emotional, or relational. This means that they are the targets of bullying and experience the physical, emotional, or relational violence directed towards them from perpetrators of bullying incidents.

Across developmental stages, the probability of being victimized in addition to the type of victimization varies [18]. Research reveals the prevalence of peer victimization as cause for alarm particularly because this manifests even among elementary school students. Elementary students are more likely to experience peer victimization from physical bullying than are adolescents in higher grades [18,19]. However, research continues to emerge showing that youth of all ages are at risk of victimization by peers in schools [7,20,21].

Several scholars have demonstrated a positive association between peer victimization and the internalizing symptoms of anxiety and depression [22,23]. For example, [24] showed that middle school students who reported being victimized by peers were more likely to self-report symptoms of anxiety or depression [25] investigated the association between peer victimization and depression among 2586 Scottish students during early adolescence. The researchers found that victimization was associated with increased feelings of depression [26] found that increases in physical and relational victimization were related to increases in internalizing symptoms (e.g., separation anxiety, generalized anxiety, depressed mood) in a sample of adolescents across a 4-year period [27] assessed the relationship between peer victimization and social anxiety in a sample of adolescents and found that relational victimization over time was associated with increased self-reported social anxiety and social phobia symptoms.

The literature provides support to the existence of a gender-based gap in peer victimization. Specifically, boys are more often exposed to physical bullying and more likely to be victims of physical bullying than girls [28]. Girls in contrast are more likely to experience relational victimization and perpetrate it as well [29]. The patterns of internalizing symptoms also vary according to gender. Higher depression levels were reported by girls in addition to generalized anxiety and social anxiety when compared with boys, particularly in terms of relational or indirect aggression [30,31]. Like gender differences, racial differences also manifest in patterns of peer victimization along with internalizing symptoms [32,33]. According to [31] in a sample of early elementary schoolers the risk of peer victimization was higher for African Americans than Hispanic and White students. Researchers also found that White and African American students were more likely to encounter repeated incidents of peer victimization when compared to Hispanic counterparts. Likewise, in terms of the type of victimization experienced, African American youth were more likely to experience direct victimization than relational or indirect victimization [34]. Other research has suggested that the context of the school environment matters, particularly, the racial makeup of the classroom. Students of color face more peer victimization when in ethnically diverse classrooms according to [35].

Beyond this, perceptions of school climate and student performance have been linked to peer victimization and internalizing symptoms. For example [36], demonstrated that victims of bullying during early adolescence perceived their schools as unsafe, yet they did not feel mistreated by teachers or administrators. These victims also had lower GPAs and school engagement in comparison to the normative group. In addition, compared to the normative group, victims of bullying were more depressed, anxious, and lonely and reported lower self-esteem [36].

### 2.2. School Connectedness as a Mediator

School connectedness is an underemphasized protective factor in adolescent mental health, specifically with regard to depression and anxiety symptoms [37]. Although primarily investigated as a critical factor in school and academic outcomes [38,39,40,41], school connectedness has been linked to adolescent mental health well-being [42,43,44,45]. School connectedness is characterized as students feeling psychologically attached or identifying with the school community [46]. This construct is often referred to by many other terms (e.g., school membership, school bonding, school belonging, school climate), demonstrating its multidimensional nature and lack of consensus in the field of education [47]. Notably, school connectedness has been found to buffer against internalizing symptoms (e.g., depression) among economically disadvantaged youth [48]. The psychological sense of connecting with the school environment is argued to be particularly important for adolescents as they rely less on family as part of their development and come to rely more on relationships that are usually found in schools or with peers [46,49,50].

Drawing from the self-determination theory [51], three central psychological needs including competence, relatedness, and autonomy are crucial in determining personal well-being. As applied to school settings, researchers emphasize that well-being can be raised through school connectedness through a process of relatedness and the desire to attach and connect to others in school [52,53]. From this prospective, it is hypothesized that school connectedness mediates, or accounts for the effects of peer victimization on adolescent mental health well-being. Prior research has directly tested the mediational role of school connectedness on adolescent mental health. For example [10], found that the relationship between peer victimization and mental well-being was mediated by school connectedness among Pakistani adolescents with an average age of 16.1 years. Similarly [11], found that exposure to peer victimization was associated with increased depressive symptoms in a sample of majority African American (44.3%) middle school students. In addition, the researchers found that school belonging mediated long-term problems that were related to peer victimization via reductions in depressive symptoms. Ref. [12] examined the relations among peer victimization, school belonging, and mental health with an ethnically diverse sample of transgender adolescents. The researchers showed that school belonging was associated with improved mental health outcomes and mediated the relationship between victimization and mental health issues (e.g., depression, suicidal ideation).

### 2.3. Race as a Moderator

Research on the role of race as a moderating variable in understanding the associations among peer victimization, school connectedness, and adolescent mental health is limited. One area of exploration has involved the study of transgendered youth of color. For example [12], examined the moderating effect of race on the mediational relationship between peer victimization, school belonging, and mental health outcomes. The results revealed that being a transgender youth of color did not necessarily differentiate the negative impact of peer victimization on mental health and belonging. Similarly, [54] examined the relationships among peer victimization, school belonging, and substance abuse among high school teenagers. Their results showed that transgendered youth of color did not report greater drug use than their White peers, despite experiencing more victimization [54]. In sum, more research is needed to contribute to the current literature and to understand the effects of race on the relationships between peer victimization, school connectedness, and mental health outcomes in a diverse sample of economically disadvantaged youth.

### 2.4. Theoretical Framework

This current study draws on concepts from the self-determination theory for explanatory purposes. Self-determination theory (SDT) is a psychological theory of innate needs that are essential for facilitating optimal functioning and personal well-being. The three basic needs include autonomy, competence, and relatedness to others. Autonomy emphasizes the need to feel in control of one’s own behaviors. Competence is characterized as the ability to feel mastery of skills for an activity. Relatedness is a universal need to experience a sense of connection or attachment to other people [51]. Social environments that allow satisfaction of these needs are theorized to promote psychological health and well-being [51,55]. Consequently, school environments are likely to impact adolescent mental health well-being through students’ perceptions of school connectedness as a process of relatedness and the desire to attach and connect to others [52,53].

## 3. Present Study

**Figure 1:**
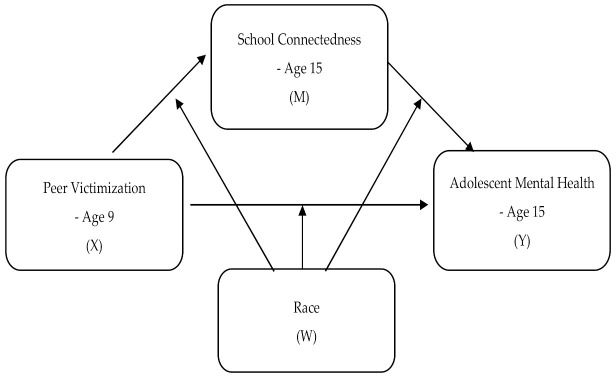
Proposed moderated mediation model.

The primary aims of the present study were to investigate whether peer victimization is associated with internalizing problems among adolescents over a 6-year period, and whether school connectedness mediates this link. In addition, this study tested whether this mediation process was moderated by race. The research objectives form a moderated mediation model (see Figure 1), which can address both mediation (i.e., how does peer victimization link to internalizing problems) and moderation (i.e., when is the link most potent) mechanisms underlying the relationship between peer victimization and internalizing problems. Based on theoretical and empirical grounds, the following hypotheses were proposed:

**Hypothesis 1 (H1):**  *There will be a positive relationship between peer victimization and teen depression and teen anxiety.*

**Hypothesis 2 (H2):**  *School connectedness would mediate the association between peer victimization and teen depression.*

**Hypothesis 3 (H3):**  *School connectedness would mediate the association between peer victimization and teen anxiety.*

**Hypothesis 4 (H4):**  *Race would moderate the direct and indirect effects of peer victimization on teen depression via school connectedness.*

**Hypothesis 5 (H5):**  *Race would moderate the direct and indirect effects of peer victimization on teen anxiety via school connectedness.*

## 4. Methods

### 4.1. Data Source and Sample

The data used for analysis are from the Fragile Families and Child Wellbeing Study (FFCWS). This longitudinal birth cohort study investigates the well-being of majority low-income, unwed parents and the health development of their children. Through multistage stratified random sampling, it follows nearly 5000 children who were born between 1998 and 2000 in 20 large U.S. cities (with populations over 200,000 in 1994), as well as their parents, with an oversampling of nonmarital births [56]. Data collection has consisted of six waves so far with interviews of mothers, fathers, and/or primary caregivers at the child’s birth and at ages 1, 3, 5, 9, and 15; in-home assessments at ages 3, 5, 9, and 15; and child surveys at ages 9 and 15 [57]. The data from age 9 and 15 child surveys were used for the current longitudinal study. This dataset provides a unique opportunity to understand how children born into these families fare with a focus on educational experiences and mental health outcomes.

Variables used for the analyses come from the public-use FFCWS dataset and they can be freely downloaded from Princeton University’s Office of Population Research data archive [57]. The analytic sample consisted of 2467 youth after selecting cases based on participation in both Year 9 and Year 15 youth interviews and handling missing data on key study variables including peer victimization, teen depression, teen anxiety, and school connectedness. The sample was equally divided between male and female with an average age of 15.50 years (*SD* = 0.69). In terms of race/ethnicity, 18.2% identified as White, non-Hispanic and 81.8% identified as either Black/African American, non-Hispanic (54.8%) or Hispanic/Latino (27%). Almost half of the sample (49.5%) were from low-income households.

### 4.2. Measures

All variables included in the analyses were from FFCWS database and instruments consisted of primary caregiver and child surveys. Wave 5 data collection period lasted from 2007–2010, around child’s 9th birthday (Year 9) and wave 6 data collection period was from 2014–2017 around child’s 15th birthday (Year 15) [57].

#### 4.2.1. Peer Victimization

Peer victimization was assessed using four items adopted from the Panel Study of Income Dynamics Child Development Supplement (PSID-CDS-III) [58]. The four-item scale asked youth at age 9 how often in the last month did kids in their school “pick on them or say mean things.” “hit or threaten to hurt them,” “take things, like money or lunch,” and “purposely leave them out.” Responses ranged from 0 (*never*) to 4 (*about every day*). Item scores were summed and served as the final composite measure. Scores ranged from 0–16, with higher values indicating greater peer victimization [57]. The Cronbach’s alpha coefficient for the current sample was 0.68.

#### 4.2.2. Teen Depression

Teen depression was assessed using five items adopted from the Center for Epidemiologic Studies Depression Scale (CES-D) [59]. The five-item scale asked youth at age 15 how often in the last month did you “feel you could not shake off the blues even with help,” “feel sad,” “feel happy,” “felt life was not worth living,” and “felt depressed.” Responses ranged from 1 (*strongly agree*) to 4 (*strongly disagree*). Items were reversed scored and the sum of the five items served as the final composite measure. Scores ranged from 5–20, with higher values indicating greater feelings of depression [57]. The Cronbach’s alpha coefficient for the current sample was 0.76.

#### 4.2.3. Teen Anxiety

Teen anxiety was assessed using six items adopted from the Brief Symptom Inventory 18 (BSI 18) [60]. The six-item scale asked youth at age 15 how often in the last month did you “have spells of terror or panic,” “feel tense,” “feel nervous,” “feel fearful,” “get suddenly scared for no reason,” and “feel restless.” Responses ranged from 1 (*strongly agree*) to 4 (*strongly disagree*). Items were reversed scored and the sum of the six items served as the final composite measure. Scores ranged from 6–24, with higher values indicating greater feelings of anxiety [57]. The Cronbach’s alpha coefficient for the current sample was 0.76.

#### 4.2.4. School Connectedness

Four items, measuring youth’s perception of inclusiveness, closeness, happiness, and safety at school, were modified from the PSID-CDS-III [58] to exam school connectedness at age 15. These four items (i.e., “feel a part of school,” “feel close to people at school,” “feel happy to be at school,” and “feel safe at school”) were rated on a four-point Likert scale ranging from 1 (*strongly agree*) to 4 (*strongly disagree*). Items were reversed scored and the sum of the four items served as the final composite measure. Scores ranged from 4–16, with higher values representing greater school connection [57]. The Cronbach’s alpha coefficient for the current sample was 0.73.

#### 4.2.5. Race

Race was a categorical variable that characterized teen’s race and ethnicity. Response categories included 1 for White, non-Hispanic, 2 Black/African American, non-Hispanic, and 3 Hispanic/Latino. This variable was recoded into a dummy variable (0 = White, non-Hispanic, 1 = Non-White). The Non-White category consisted of Black/African American, non-Hispanic and Hispanic/Latino youth.

#### 4.2.6. Covariates

Gender, age, family household income, academic performance, and school climate perceptions were included as statistical controls given they might confound the associations of interested variables in the current study [61]. Gender was measured as a binary variable (0 = female, 1 = male). Age was a continuous variable that measured youths’ age at Year 15 and ranged from 14–18 years. Family household income was measured using primary caregivers’ household income at Year 15. This variable was recoded 0 for low income (e.g., income ≤ 40,000) and 1 for high income (e.g., income > 40,000). Academic performance was measured using math grades at the most recent grading period during Year 15. This variable was recoded 0 for low performing (e.g., letter grade of C or lower) and 1 for high performing (e.g., letter grade of A or B).

School climate perceptions was measured by a scale that combined items from [62] and questions created by the developers of FFCWS in the Year 15 child survey. The scale consisted of 10 items that measured teaching quality and student behavior in school. Youth were asked to report their feelings on these statements: “teachers in school really care about students,” “teachers treat students with respect,” “teachers accept nothing less than full effort,” “teachers make lessons interesting,” “teachers explain difficult things clearly,” “in class we learn a lot every day,” “in class we stay busy and don’t waste time,” “kids in this school treat teachers with respect,” “kids in this school work hard,” and “kids in this school behave the way the teachers want them to.” Responses ranged from 1 (*strongly agree*) to 4 (*strongly disagree*). Items were reversed scored and the sum of all items served as the final composite measure. Scores ranged from 10–40, with higher values representing more positive perceptions of school climate [57]. The Cronbach’s alpha coefficient for the current sample was 0.85.

### 4.3. Data Analysis

Missing data patterns were examined and found to be missing at random. As a result, this study used list-wise deletion to address missing data in that all variables had less than 5% missing observations, except for peer victimization (12.4%). Cases with missing data on this key study variable were also excluded from analysis to produce unbiased estimates [63]. In addition, normal distribution of study variables were examined. The skewness and kurtosis of peer victimization, school connectedness, teen depression, and teen anxiety fell within the acceptable range (skewness cutoff of 2.0 and kurtosis cutoff of 9.0; [64]). Data analytic strategies included the use of descriptive, bivariate, and multivariate techniques. Descriptive statistics (means and standard deviations for continuous variables and percentages for categorical variables) were used to delineate all the variables included in the analysis. Next, bivariate correlations among study variables were assessed [H1].

For the primary analysis, [65] PROCESS macro (Model 4) was used to test the mediating effect of school connectedness on teen depression [H2] and teen anxiety [H3]. Two sets of mediational analyses were performed. This macro uses bootstrapping method to examine the mediating effect and it performs better (both in terms of its statistical power and Type I error) than the traditional causal steps approach. Specifically, bootstrapping method produced 95% bias-corrected confidence intervals for the indirect effect from 5000 resamples of the data. Mediation was deemed to be statistically significant if the confidence intervals did not include zero. In addition, using two sets of moderated mediation analyses, the study further explored whether each mediation process was moderated by race [H4 and H5]. The analysis of the moderated mediation models were performed using Hayes’s PROCESS macro (Model 59) [65]. All analyses were conducted using SPSS 27 [66]. Gender, age, family household income, academic performance, and school climate perceptions were included in all models as statistical controls.

## 5. Results

### 5.1. Descriptive Information

The descriptive statistics and correlations for study variables are presented in Table 1. Among the 2467 adolescents examined, 73% reported experiencing a depressive symptom and 86% reported experiencing at least one symptom of anxiety within the past month. The average teen depression and anxiety score was 7.96 (*SD* = 2.99) and 10.85 (*SD* = 3.91), respectively. In regard to academic performance, 39% of the sample were low performing students. For White students, the average depression and anxiety score was 7.73 (*SD =* 3.07) and 10.76 (*SD* = 4.09), respectively. For Non-White students, the average depression and anxiety score was 8.01 (*SD =* 2.98) and 10.87 (*SD* = 3.87), respectively. The average peer victimization score for White students was 2.24 (*SD* = 2.76) and 2.43 (*SD* = 3.09) for Non-White students. The average school connectedness score for White students was 14.17 (*SD* = 2.09) and 13.65 (*SD* = 2.30) for Non-White students.

### 5.2. Correlations

As shown in Table 1, peer victimization was significantly and positively associated with teen depression (*r* = 0.134, *p* < 0.01) and teen anxiety (*r* = 0.145, *p* < 0.01) over a 6-year period, thus supporting H1. Also, school connectedness was negatively associated with race (*r* = −0.087, *p* < 0.01), peer victimization (*r* = −0.107, *p* < 0.01), teen depression (*r* = −0.354, *p* < 0.01), and teen anxiety (*r* = −0.220, *p* < 0.01).

### 5.3. Mediation Model [H2]

**Figure 2:**
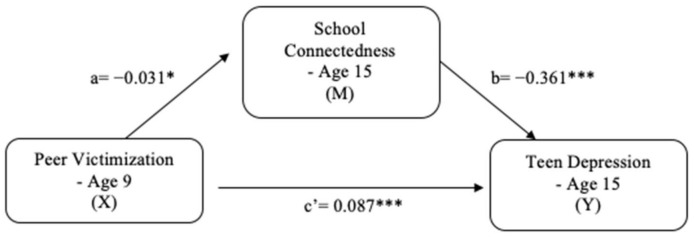
Mediation model for teen depression. * *p* < 0.05; *** *p* < 0.001. Note: The path coefficients (a, b, c’) estimate the strength of the hypothesized associations using unstandardized regression coefficients; a = the direct effect of X on M; b = the direct effect of M on Y; c’ = the direct effect of X on Y.

As shown in Figure 2, the mediation model examined the direct and indirect effects of peer victimization on teen depression through school connectedness. The significant direct effects in the mediation model are presented: (a) peer victimization was significantly and negatively associated with school connectedness (*b* = −0.031, *p* = 0.012), (b) school connectedness was significantly and negatively associated with teen depression (*b* = −0.361, *p* < 0.001), and (c’) peer victimization was significantly and positively associated with teen depression (*b* = 0.087, *p* < 0.001). Furthermore, peer victimization had a significant indirect effect on teen depression through school connectedness (*b* = 0.011, 95% CI [0.002, 0.021]), and the indirect effect’s proportion of the total effect was 11%. The results indicate that school connectedness has a mediating effect on the relationship between peer victimization and teen depression, thus supporting H2.

### 5.4. Mediation Model [H3]

**Figure 3:**
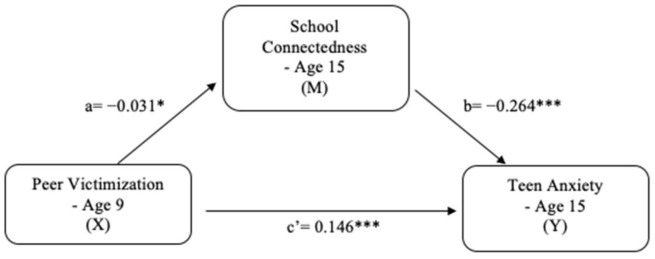
Mediation model for teen anxiety. * *p* < 0.05; *** *p* < 0.001. Note: The path coefficients (a, b, c’) estimate the strength of the hypothesized associations using unstandardized regression coefficients; a = the direct effect of X on M; b = the direct effect of M on Y; c’ = the direct effect of X on Y.

As shown in Figure 3, the mediation model examined the direct and indirect effects of peer victimization on teen anxiety through school connectedness. The significant direct effects in the mediation model are presented: (a) peer victimization was significantly and negatively associated with school connectedness (*b* = −0.031, *p* = 0.012), (b) school connectedness was significantly and negatively associated with teen anxiety (*b* = −0.264, *p* < 0.001), and (c’) peer victimization was significantly and positively associated with teen anxiety (*b* = 0.146, *p* < 0.001). Furthermore, peer victimization had a significant indirect effect on teen anxiety through school connectedness (*b* = 0.008, 95% CI [0.001, 0.016]), and the indirect effect’s proportion of the total effect was 5%. The results indicate that school connectedness has a mediating effect on the relationship between peer victimization and teen anxiety, thus supporting H3.

### 5.5. Moderated Mediation Model [H4]

**Figure 4:**
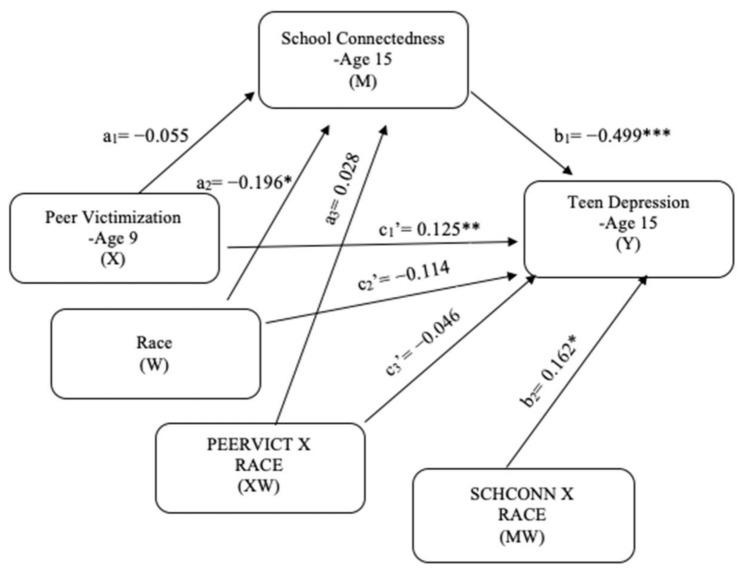
Moderated mediation model for teen depression. * *p* < 0.05; ** *p* < 0.01; *** *p* < 0.001.

**Figure 5:**
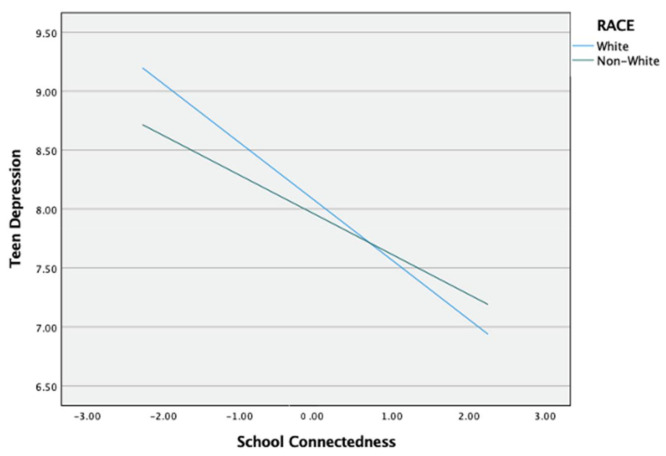
Moderation effect of race in the relationship between peer victimization and teen depression.

The moderated mediation results for teen depression (see Figure 4) showed a significant interaction effect of race on the direct effect of school connectedness and teen depression (*b* = 0.162, *p* = 0.018). For descriptive purpose, school connectedness was plotted against teen depression separately for White and Non-White students (Figure 5). Simple slope tests revealed that both White and Non-White students with high school connectedness had significantly lower teen depression. However, this effect was weaker for Non-White students (b_simple_ = −0.337, *p* < 0.001) than for White students (b_simple_ = −0.499, *p* < 0.001). There was not a significant moderating effect of race on the direct effect of peer victimization and school connectedness and peer victimization and teen depression (see Table 2). To test the influence of race on the mediated relationship between peer victimization and teen depression, the index of moderated mediation was used. The index of moderated mediation, which is a direct quantification of equality of the conditional indirect effect for moderation, was not significant because its confidence interval included 0 (*b* = −0.018, 95% CI [−0.053, 0.015]). This indicates that race does not moderate the indirect effect of school connectedness on the relationship between peer victimization and teen depression. Given that race moderated just the second-stage moderation model (b-path), which is a type of moderated mediation model established by [65], H4 was partially supported.

### 5.6. Moderated Mediation Model [H5]

**Figure 6:**
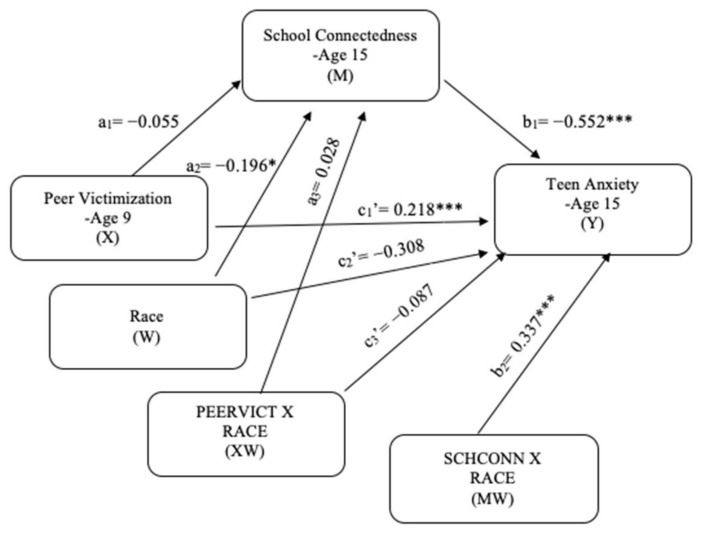
Moderated mediation model for teen anxiety. * *p* < 0.05; *** *p* < 0.001.

**Figure 7:**
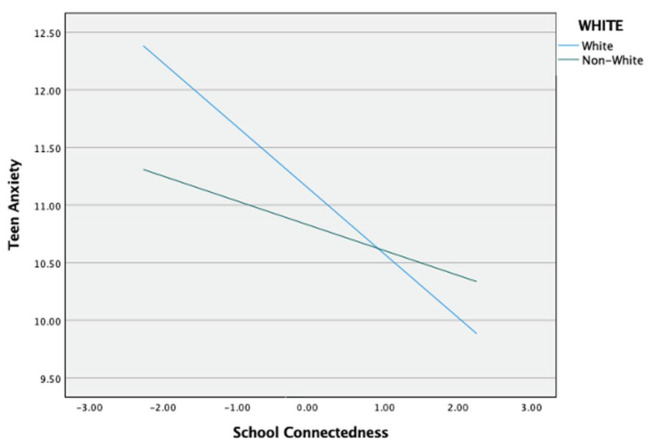
Moderation effect of race in the relationship between peer victimization and teen anxiety.

The moderated mediation results for teen anxiety (see Figure 6) showed a significant interaction effect of race on the direct effect of school connectedness and teen anxiety (*b* = 0.337, *p* < 0.001). For descriptive purpose, school connectedness was plotted against teen anxiety separately for White and Non-White students (Figure 7). Simple slope tests revealed that both White and Non-White students with high school connectedness had significantly lower teen anxiety. However, this effect was weaker for Non-White students (b_simple_ = −0.215, *p* < 0.001) than for White students (b_simple_ = *b* = −0.552, *p* < 0.001). There was not a significant moderating effect of race on the direct effect of peer victimization and school connectedness and peer victimization and teen anxiety (see Table 3). The index of moderated mediation was not significant because its confidence interval included 0 (*b* = −0.025, 95% CI [−0.068, 0.010]). This indicates that race does not moderate the indirect effect of school connectedness on the relationship between peer victimization and teen anxiety. Given that race moderated just the second-stage moderation model, H5 was partially supported.

## 6. Discussion

Empirical support exists for a link between peer victimization and internalizing symptoms of anxiety and depression among school-age youth, in which, victimization positively predicts internalizing problems [7,22,23]. However, much less is known about the mediating and moderating mechanisms underlying this relationship. The purpose of this study was to investigate whether peer victimization at age 9 indirectly related to internalizing problems at age 15 via school connectedness and whether the direct and indirect associations between peer victimization and internalizing problems were moderated by race. After controlling for gender, age, family household income, academic performance, and school climate perceptions, findings revealed that peer victimization at age 9 was positively associated with internalizing problems at age 15, which is consistent with previous research. Beyond this, the findings indicated that the predictive effect of peer victimization over a 6-year period on teen depression and anxiety was explained by increased school connectedness. Furthermore, there was a moderating effect of race on the direct effect of school connectedness and teen depression and anxiety. For both White and ethnic minority students, increased school connectedness was associated with less teen depression and anxiety. However, this effect was weaker for ethnic minority students in comparison to White students in both moderated mediation models. The moderated mediation results for teen anxiety showed a greater differential effect among race.

### 6.1. The Mediating Role of School Connectedness

Previous literature surrounding adolescent mental health has taken on a risk perspective, leaving practitioners and policy makers with an understanding of the negative causal influences [67,68]. This approach negates the role of protective factors as a way to mitigate risks. The current study advances our understanding of early exposure to victimization and its association with internalizing problems, and the role of school connectedness as an essential protective factor. Consistent with previous research investigating effects of victimization [10,69,70], this study provides support for school connectedness as an underlying mechanism mediating the effect of victimization on teen depression and anxiety. The mediation analysis demonstrated that peer victimization predicted diminished school connectedness and that an increased sense of school connectedness was associated with less teen depression and anxiety. Although school connectedness demonstrated protective qualities, the mediating effect was minimal in both moderated mediation models. This finding may be due to some other underlying mechanisms such as peer support explaining this association. Because no other intermediate variables were analyzed, other factors in this indirect path of depression and anxiety could not be compared. Future research should explore multiple mediating factors simultaneously. Beyond this, this study does provide support for the self-determination theory [51] and the psychological need for relatedness. From this perspective, school connectedness is a social and interpersonal phenomenon, in which, youth have an innate desire to attach and connect to others in the school setting which in turn improves overall mental well-being.

### 6.2. The Moderating Role of Race

This study also contributes to the literature by expanding on the mediating models to include the moderating effect of race. Thus, the differential relations between peer victimization and internalizing problems were tested across White and Non-White students. There is empirical support that suggests a sense of school connection is essential for most youth, and even more important for youth that are economically disadvantaged and ethnic minorities [54,71]. This conditional process analysis was necessary in understanding the effect of racial differences. The study identified a second-stage moderation regarding race and teen depression and anxiety, which is contradictory to research that found no moderating effects of race on victimization and mental health outcomes via school belonging [12,54]. Findings showed that race moderated the direct effect of school connectedness and teen depression and anxiety. For both White and ethnic minority students, increased school connectedness was associated with less teen depression and anxiety. However, this effect was weaker for students of color in comparison to White students in both conditional models. This could be due to White students on average having a greater sense of school connection in comparison to youth of color [72] and as a result the association between school connectedness and teen depression and anxiety has a greater impact on White students, in terms of fewer internalizing symptoms, than students of color. This finding has the potential to inform the enhancement of targeted school-based interventions and prevention efforts.

An examination of the moderation of the relationship between peer victimization and school connectedness and peer victimization and mental health offered important insights. Findings demonstrated that although ethnic minority youth were at risk for more victimization and therefore more internalizing problems and less school connectedness, White students were just as likely to be victims of bullying incidents. The inconsistency in experiences of victimization across racial groups have led researchers to conclude that race alone is not directly related to peer victimization [73]. One area of exploration to account for bullying experiences is the intersectionality of the racial composition of the school/classroom and the race of victims [35,74]. Theories such as the imbalance of power thesis [75] and group threat theory [76] explain the dynamics of intergroup racial contact and potential contribution to peer victimization.

## 7. Limitations and Future Directions

The study contributes to the understanding of early exposure to peer victimization and its association with internalizing problems and when this relationship varied among race. Several limitations, however, should be addressed. First, data were collected through self-report measures. Relying on the self-reporting of participants runs the risk of reporter bias. For example, surveys that assess highly sensitive risk behaviors like bullying behaviors and mental health symptomology may be underreported due to social desirability. Future studies should include more objective measures and other report measures from different informants (e.g., parent or teacher). Second, the measurement of peer victimization was limited and did not include the effects of cyberbullying. Future research should explore the impacts of cyberbullying and its relationship to mental health. Third, this study was not able to control for the initial effects of depression and anxiety at Year 9 because these measures were not available in the dataset. This is important and needed in future research. A final limitation is that this study relied on only two assessment waves of targeted constructs. Future research should focus on the inclusion of three or more assessment points providing greater information on the pattern of change over time, as well as the opportunity to use growth curve modeling to test relationships between peer victimization and internalizing problems. In addition, research that employs a nationally representative sample and that could offer a broader understanding of the relationships among peer victimization, school connectedness, and internalizing problems will extend these findings considerably. Further, research that could provide important contextual information about the schools that are most successful in cultivating a sense of school connection for students of color will have important implications for educational policy.

## 8. Implications

Despite these limitations, the findings in the current study have important practical and research implications.

### 8.1. School Implications

This study provides promising evidence that school connectedness may be an important factor for decreasing internalizing symptoms among victims of bullying, particularly ethnic minority students. School administrators and school staff including school social workers, school counselors, and school psychologist should work to create inclusive and supportive school climates that encourage students of color to form strong connections and to feel safe and welcomed. The push for increased connectedness, leading toward improvement in school members’ experiences of safety and decreased internalizing problems could take on many forms. For example, school staff could seek out climate-building programs with components that directly target school connectedness. Administrators and other officials could monitor student connectedness as a more proximal outcome of school climate intervention efforts. Additional examples include the enactment of school policies to ensure that students of color feel psychologically attached to school and accepted and the creation and support of clubs that engage ethnic minority youth experiencing mental health symptomology. Beyond this, school mental health providers must consider school connectedness as an underlying factor in overall student mental health assessment and treatment. In terms of professional development, school mental health professionals can offer teachers training on understanding the importance of how their behavior impacts school connectedness, particularly for students of color. Similarly, professional development and education of teachers can demonstrate how to effectively engage students beyond the daily activities of learning. 

### 8.2. Research Implications

An important research implication of this study is the crucial knowledge gap regarding the weaker school connectedness levels of students of color. Given that the majority of students in public schools are students of color, the findings that the moderating relationship between school connectedness and internalizing problems is weaker for students of color needs to be further explored from a racial justice perspective. Researchers have the opportunity to ask why this weaker relationship exists and how it ultimately influences the mental health of students of color. Additionally, school connectedness in the era of COVID-19 is an understudied topic. Like the health research on COVID-19 has demonstrated gross racial disparities in the disease and death rates, research of the racial differences in school connectedness during COVID-19 will be essential to prioritizing the mental health outcomes and treatment of students in schools. 

## 9. Conclusions

School officials must continue to prioritize efforts and activities that increase student connectedness as they target a reduction of internalizing symptoms in adolescents who attend their schools. This study demonstrates the importance of school connectedness as a mediator of internalizing problems in adolescents. School connectedness is especially important as student relationships are in flux due to the global pandemic. Even as schools eventually regain normalcy, they can bolster efforts to increase school connectedness for all students, with extra attention to this metric for students of color. 

## Figures and Tables

**Table 1:** Descriptive statistics and correlations among variables (*n* = 2467).

Variable	% or M (SD)	1	2	3	4	5	6	7	8	9	10
1. Peer Victimization	2.40 (3.05)	----									
2. Teen Depression	7.96 (3.00)	0.134 **	-----								
3. Teen Anxiety	10.85 (3.91)	0.145 **	0.652 **	-----							
4. School Connectedness	13.75 (2.27)	−0.107 **	−0.354 **	−0.220 **	-----						
5. Race		0.024	0.035	0.011	−0.087 **	-----					
White [reference]	18%										
Non-White	82%										
6. Gender		0.011	−0.122 **	−0.084 **	0.094 **	0.009	-----				
Female [reference]	50%										
Male	50%										
7. Age	15.50 (0.69)	−0.030	0.010	−0.019	−0.051 *	0.110 **	0.014	-----			
8. Family household income		−0.090 **	−0.085 **	−0.066 **	0.121 **	−0.239 **	0.022	−0.040 *	-----		
Low ≤ 40,000 [reference]	50%										
High > 40,000	50%										
9. Academic Performance		−0.088 **	−0.111 **	−0.093 **	0.110 **	−0.081 **	−0.060*	−0.030	0.080 **	-----	
Low [reference]	39%										
High	61%										
10. School Climate Perceptions	31.44 (5.15)	−0.097 **	−0.269 **	−0.178 **	0.590 **	−0.046*	0.050 *	−0.049 *	0.020	0.128 **	-----

Note: % = percentage for categorical variables; M (SD) = mean (standard deviation) for continuous variables. * *p* < 0.05; ** *p* < 0.01.

**Table 2:** Testing the moderated mediation effect of peer victimization on teen depression (*n* = 2467).

Variable	M (School Connectedness)		Y (Teen Depression)	
	*b*	*SE*	*b*	*SE*
Gender	0.297 ***	0.073	−0.568 ***	0.112
Age	−0.058	0.054	−0.030	0.082
Academic Performance	0.117	0.076	−0.394 ***	0.116
School Climate Perceptions	0.253 ***	0.007	−0.049 ***	0.013
Household Income	0.427 ***	0.076	−0.220	0.116
Peer Victimization (X)	−0.055	0.031	0.125 **	0.048
Race (W)	−0.196 *	0.098	−0.114	0.151
Peer Victimization × Race	0.028	0.034	−0.046	0.052
School Connectedness (M)			−0.499 ***	0.066
School Connectedness × Race			0.162 *	0.069
*R* ^2^	0.368 ***		0.156 ***	
*F*	178.71		45.47	
				
Index of Moderated Mediation	Index	BootSE	BootLLCI	BootULCI
PEERVICT → SCHCON → TDEPR	−0.018	0.017	−0.053	0.015

Note: For gender, 0 = female, 1 male; for race, 0 = White, 1 = Non-White; for academic performance, 0 = low, 1 = high; for household income, 0 = low, 1 = high; PEERVICT = peer victimization, SCHCON = school connectedness; TDEPR = teen depression. * *p* < 0.05; ** *p* < 0.01; *** *p* < 0.001.

**Table 3:** Testing the moderated mediation effect of peer victimization on teen anxiety (*n* = 2467).

Variable	M (School Connectedness)		Y (Teen Anxiety)	
	*b*	*SE*	*b*	*SE*
Gender	0.297 ***	0.073	−0.524 ***	0.152
Age	−0.058	0.054	−0.155	0.111
Academic Performance	0.117	0.076	−0.467 **	0.158
School Climate Perceptions	0.253 ***	0.007	−0.049 **	0.018
Household Income	0.427 ***	0.076	−0.271	0.158
Peer Victimization (X)	−0.055	0.031	0.218 ***	0.065
Race (W)	−0.196 *	0.098	−0.308	0.206
Peer Victimization × Race	0.028	0.034	−0.087	0.071
School Connectedness (M)			−0.552 ***	0.090
School Connectedness × Race			0.337 ***	0.093
*R* ^2^	0.368 ***		0.082 ***	
*F*	178.71		21.99	
Index of Moderated Mediation	Index	BootSE	BootLLCI	BootULCI
PEERVICT → SCHCON → TANX	−0.025	0.020	−0.068	0.010

Note: For gender, 0 = female, 1 male; for race, 0 = White, 1 = Non-White; for academic performance, 0 = low, 1 = high; for household income, 0 = low, 1 = high; PEERVICT = peer victimization, SCHCON = school connectedness; TANX = teen anxiety. * *p* < 0.05; ** *p* < 0.01; *** *p* < 0.001.

## Data Availability

This research is based on public data from the Fragile Families and Child Wellbeing Study (FFCWS) provided by Princeton University’s Center for Research on Child Wellbeing (CRCW) and the Columbia Population Research Center (CPRC). This data can be found here: https://fragilefamilies.princeton.edu/documentation. The views and opinions expressed in this paper are those of the author and do not represent the views of CRCW and CPRC.
